# Promising Nanoparticle-Based Heat Transfer Fluids—Environmental and Techno-Economic Analysis Compared to Conventional Fluids

**DOI:** 10.3390/ijms22179201

**Published:** 2021-08-25

**Authors:** Natalia Czaplicka, Anna Grzegórska, Jan Wajs, Joanna Sobczak, Andrzej Rogala

**Affiliations:** 1 Department of Process Engineering and Chemical Technology, Faculty of Chemistry, Gdansk University of Technology, Narutowicza 11/12, 80-233 Gdansk, Poland; anna.grzegorska@pg.edu.pl (A.G.); andrzej.rogala@pg.edu.pl (A.R.); 2 Institute of Energy, Faculty of Mechanical Engineering and Ship Technology, Gdansk University of Technology, Narutowicza 11/12, 80-233 Gdansk, Poland; jan.wajs@pg.edu.pl; 3 Research and Development Joanna Sobczak, Różnowo 8, 14-240 Susz, Poland; joasobczak@outlook.com

**Keywords:** nanoparticles, nanofluids, heat transfer fluids, heating and cooling systems

## Abstract

Providing optimal operating conditions is one of the major challenges for effective heating or cooling systems. Moreover, proper adjustment of the heat transfer fluid is also important from the viewpoint of the correct operation, maintenance, and cost efficiency of these systems. Therefore, in this paper, a detailed review of recent work on the subject of conventional and novel heat transfer fluid applications is presented. Particular attention is paid to the novel nanoparticle-based materials used as heat transfer fluids. In-depth comparison of environmental, technical, and economic characteristics is discussed. Thermophysical properties including thermal conductivity, specific heat, density, viscosity, and Prandtl number are compared. Furthermore, the possible benefits and limitations of various transfer fluids in the fields of application are taken into account.

## 1. Introduction

Providing optimal operating conditions is one of the major challenges for effective heating or cooling systems. The great importance of these systems is to enhance the rate of heat or cold transfer by the application of advanced working fluids. However, several operation and maintenance key criteria must be met when the usage of transfer fluids is considered. Heat transfer fluids (HTFs) are widely used in many industrial and consumer applications and are characterized by two groups of parameters, physicochemical and thermal properties. Among the physicochemical parameters, the most important are kinematic viscosity, flash point, and pour point [1,2]. Viscosity determines the flow in the system, and to ensure sufficiently small temperature gradients between the heat carrier and the heat transfer surface, it is necessary to provide a turbulent flow, characterized by a Reynolds number above 2100 in linear channels with circular cross sections. The flash point determines the possibility of HTF inflammation in contact with heated surfaces. Therefore, it is important for safety, for example, in the event of leaks in the system. Furthermore, the pour point and viscosity are important for starting the system, especially in low-temperature conditions. The minimum start-up temperature should be about 5–10 °C above the pour point, or it is determined by the minimum temperature at which the fluid viscosity becomes the maximum allowable value for the pumps installed in the system. Thus, the high viscosity of the fluid is undesirable, due to the fact that this affects the lowest start-up temperature of the system and the pumping power, and the pressure loss of the system [3]. The possibility of using a given HTF in a specific heat transfer system is determined by its thermal properties, i.e., those that are taken into account in the calculations of heat transfer systems. These are density, kinematic, and dynamic viscosity in the operating temperature range, specific heat, thermal conductivity, and Prandtl number [2]. The Prandtl number is a criterion number that characterizes the similarity of the physical properties of fluids in heat transfer processes. It represents the ratio of momentum diffusivity to thermal diffusivity in a fluid. A high value of the Prandtl number means that the fluid has a high ability to transfer momentum and a low ability to transfer heat, while a low value of this number indicates a low ability to transfer momentum and a high ability to transfer heat [4].

**Figure 1:**
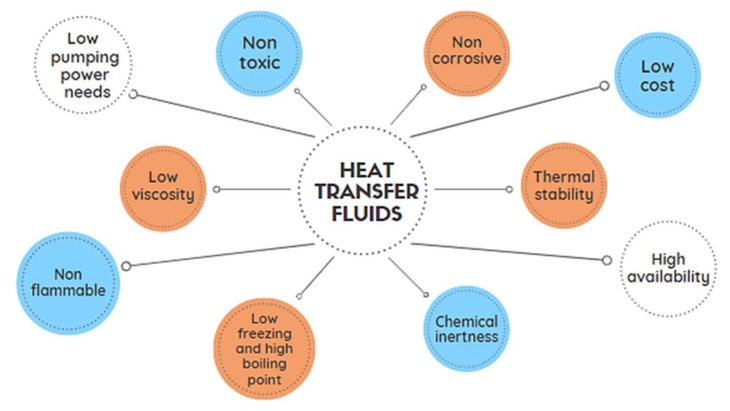
Characteristics of the ideal HTFs.

Depending on the field of technology, specific requirements are imposed on the properties of HTFs. For example, in ferrous and non-ferrous metallurgy, high thermal stability is a desirable property in HTFs, while in the space rocket industry, low density and low volatile liquids are required [5,6]. However, there are characteristics that should characterize the fluid used as HTF, regardless of the system in which it is to be applied, as shown in Figure 1. First, the HTFs must show a very good heat transfer capacity in the selected temperature range, i.e., high specific heat and a high thermal conductivity coefficient [1]. In addition, they should be characterized by high thermal and thermooxidative stability, compatibility with construction materials used in heating systems, and non-toxicity [7,8]. HTFs should not be prone to secretion and deposition of sediments on the walls of the system. It is also important to have the appropriate viscosity in the operating temperature range and the pour point adjusted to the conditions to which the system may be exposed [9]. The life charge of the fluid is also important. A good HTF is considered to have a useful life of a charge in the range of 35,000 to 40,000 effective working hours [10]. Currently, a priority trend in heat exchange technology is the search for compounds and the creation of fluid compositions that have the desired combination of key physicochemical properties to solve unconventional problems. Finally, economic features such as the availability and low cost of HTFs should be taken into account.

**Figure 2:**
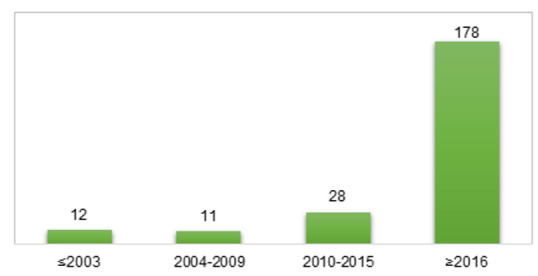
Number of articles cited in this review by year of publication.

As the topic of heating and cooling is present in practically every area of industry, it is not surprising that for years, heat transfer fluids have attracted a lot of attention. There are many reviews of heat transfer fluids in the literature that have been developed from different viewpoints. Many of them refer to HTFs used in solar power technology [1,11,12,13,14,15,16,17,18], the use of nanofluids and their modifications [19,20,21,22,23,24,25,26,27,28,29,30], and ionic liquids [5,10,31,32,33,34] as HTFs, and the analysis of commercial HTFs [35,36]. However, despite extensive research and analysis, the most popular HTFs in most industrial applications are still conventional media, and no comparison has been made between conventional HTFs and alternative media including nanoparticle-based HTFs in the context of industrial applications. Therefore, the aim of this review is to perform an in-depth comparison of the environmental, technical, and economic characteristics of traditional and nanoparticle-based heat transfer fluids. Furthermore, the possible benefits and limitations and the branch of applications of various HTFs are taken into account. This paper focuses on the latest literature reports (mainly from the last five years) on novel HTFs and compares them with commonly and widely used traditional media. Thanks to this, it is possible to highlight the existing problems in the field of industrial HTFs, as well as to determine the direction in which research on new solutions should go to solve the current limitations. The number of articles cited in our work by years of publication is presented in Figure 2.

## 2. Conventional HTFs

**Figure 3:**
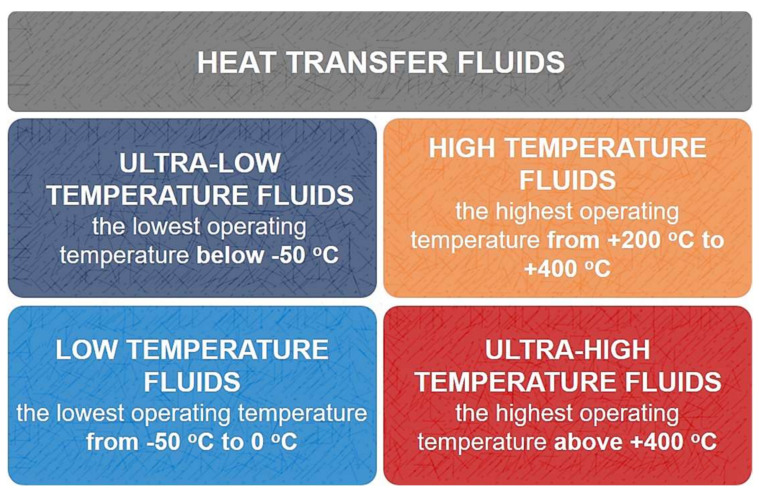
Basic categories of HTFs and their operating temperatures.

Both liquid and gaseous heat transfer fluids are commonly used, which can be classified into several categories. In terms of the specific applications of HTFs, the classification according to the operating temperature range seems most appropriate. Then, four basic categories of liquids are distinguished [9], which are presented in Figure 3. The most common industrial heat transfer fluids are oil heat carriers, including deeply refined mineral oils with added inhibitors, synthetic polyolefin-type hydrocarbons, aromatic hydrocarbons without alkyl side chains, esters of carboxylic acids and polyhydric alcohols, silicones, silicate esters and alkyl borates [6,37]. Synthetic oils, such as Therminol^®^ VP-1 or Dowtherm^®^ A, offer the widest temperature range in which they can be used [38]. Nevertheless, they are toxic and highly flammable, which poses a threat to both plant operators and the environment [39]. In addition, they have high vapor pressures that significantly exceed the atmospheric pressure. This makes their use as thermal storage media difficult due to the necessity to apply impractically large pressure vessels [40]. Furthermore, heating oils as heat carriers should only be used in systems with forced circulation. Systems using only natural convection do not provide enough rapid flow to prevent local overheating and decomposition of the oil. Therefore, finding an alternative to replace the currently implemented HTFs is an important issue that allows for reducing risk, but also reducing costs and improving the efficiency of installations [36]. This chapter briefly characterizes conventional HTFs with regard to their major disadvantages, advantages, and limitations, their thermophysical properties, and the applications in which they are used.

### 2.1. Air

The first and most basic example of a heat transfer medium is air. Air has certain advantages, including its non-corrosive nature and its non-vulnerability to freezing and boiling [2,16]. Furthermore, air is practically freely available, cost-free, non-polluting and practically does not have an upper operating temperature limit [41,42,43]. Moreover, it is an important HTF from the viewpoint of environmental safety, because leakages do not cause any problems compared to other HTFs, such as molten salt spills or mineral oil spills [43,44]. However, its most significant limitation is its lower thermal conductivity (low air heat transfer rate) and volumetric heat capacity [45]. Due to the relatively low heat capacity, it could not be used for domestic water heating purposes [2]. Furthermore, air must be pressurized or fed through very large pipes. Good et al. [41] proposed the application of air as a heat transfer fluid in a commercial concentrating solar power (CSP) plant with a peak thermal capacity of 3.9 MW_th_. Furthermore, the authors claimed that the application of air as an HTF enables the application of a proven and inexpensive thermal energy storage (TES) concept based on a packed bed of rocks. Meanwhile, Li et al. performed the analysis of TES using air as the heat transfer fluid working at +500 °C. Cinoacca et al. [46] proposed the use of compressed air as a working fluid in a parabolic trough CSP plant with TES. The authors concluded that the application of compressed air allows one to increase the air temperature at the solar field inlet and to reduce the solar collector’s length. An installation with applied compressed air at 30 bar and a 1 kg/s mass flow rate with a maximum temperature equal to +580 °C produces about 170 kW of mechanical power. However, the main limitations of this technology were the low heat transfer coefficient of air compared to a liquid HTF and the need to pressurize the gas. Toro et al. [47] pointed out that air can be used as the working fluid in solar-heated CSP plants as an alternative HTF, thus reducing maintenance operations and providing the power section with a higher degree of flexibility.

### 2.2. Halogenoalkanes

Alkyl halides such as methylene chloride, trichloroethylene (TCE) and hydrofluorocarbons are commonly used HTFs with operating temperatures below −50 °C [48]. These substances are non-flammable and non-corrosive, while those containing chlorine (methylene chloride, TCE) are highly toxic [49]. Therefore, they are systematically removed from use to eliminate them. Fluorine derivatives such as hydrofluoroethers or perfluorocarbon ethers exhibit several unique properties which make them suitable for use as ultra-low temperature HTFs. First of all, they are non-toxic and non-flammable. Moreover, some of these liquids have a very low freezing point and a low viscosity at low temperatures. On the other hand, they are definitely more expensive than their substitutes. Additionally, because of the very low surface tension of these compounds, there is a risk of leakage in the installations, which increases the cost of the process. Another disadvantage of low-freezing-point fluoroalkanes is the significantly lower boiling point compared to other heat transfer fluids. These compounds can be used in the temperature range from −100 to +150 °C.

### 2.3. Water/Steam

Similarly to air, water has good physical properties to be used as a heat transfer medium [50]. The most important characteristics of water are high specific heat (4185 J/(kg·K)), availability, low viscosity, non-toxicity, and low cost [51,52]. However, the main problems with it are related to the corrosive nature and freezing below 0 °C. Furthermore, water is chemically stable at a high temperature, but under normal pressure at +100 °C, it undergoes a phase transition from the liquid to gas phase. Thus, in the temperature range from 0 to +100 °C, water is the best fluid for heat transfer. However, when it is necessary to use a heat carrier in a wider temperature range, where the lowest temperature is much lower than 0 °C, the addition of water-soluble organic compounds or inorganic salts is most often used to lower the freezing point. Then, water solutions of glycols (ethylene and propylene), alcohols (methanol, ethanol, isopropanol), or sodium, potassium, or calcium chloride, formate and acetate are used. However, the use of aqueous solutions of such substances allows the boiling point to be slightly higher than the boiling point of pure water, i.e., +100 °C. To lower the freezing point of water, salt is often added, for example, calcium chloride or sodium chloride [53]. For example, an aqueous CaCl_2_ solution with a concentration of 29.9 wt% has a freezing point of −55 °C. Such solutions are non-flammable and non-toxic. However, they are highly corrosive and at temperatures below −20 °C, the addition of salt reduces the efficiency of heat transfer efficiency compared to pure water [54].

Water has a higher specific density, heat capacity, and thermal conductivity than steam. The limitation resulting from this is the compromise between the high pressure required for high temperature operation and induced technology difficulties, such as the necessarily thick walls of pipes, which is an obstacle to heat transfer. Furthermore, the upper limits at which water can be used as a HTF in a saturated state in thermodynamic cycles are +374 °C and 221 bar, when it reaches a supercritical level. Despite many beneficial properties, pure water/steam has not often been described in the recent literature as a HTF. Montes et al. [55] compared the application of oil, water/steam and molten salts as a HTF for parabolic trough collectors, and the water/steam system has the highest nominal overall efficiency. Meanwhile, Edwards and Bindra [56] proposed the use of saturated steam as a HTF for TES in a packed bed. Furthermore, the authors performed the analysis of air as HTF under the same conditions. It was concluded that the rate of steam injection has a significant effect on the modes of heat transfer, advection, and diffusion. In the case of steam injection, the wall heat loss rate was much lower than the heat injection rate, while for hot air injection, the wall heat loss rate was comparable to the heat injection rate. This fact was related to essentially higher fractional heat losses.

### 2.4. Hydrocarbons

The first group of hydrocarbons used as ultra-low temperature HTFs are aromatic hydrocarbons. The most common is diethylbenzene, which can be used in the temperature range from −70 to +260 °C [57]. This compound has very good heat transfer characteristics and high thermal stability at low temperatures [35]. However, the main disadvantage of diethylbenzene is its toxicity. It also has an unpleasant smell that is a nuisance to the employees. Generally, low aromatic hydrocarbons have a freezing point below −80 °C and typically such compounds are used in the temperature range above −70 °C in hermetically sealed systems [58]. The second group consists of aliphatic hydrocarbons of the paraffinic and isoparaffinic type, called aliphatic compounds based on petroleum. These types of HTFs do not form dangerous degradation by-products. Most of them have a low odor and are not toxic through skin contact or ingestion. Despite these advantages, aliphatic hydrocarbons are not widely used as ultra-low-temperature heat transfer agents [59] due to their high low-temperature viscosity and their significantly lower thermal stability compared to aromatic hydrocarbons. Some of the liquids based on isoparaffin (having 12 to 14 carbons) can be used in the temperature range of −60 to +150 °C [35]. These compounds are preferred in the food and pharmaceutical industries where non-toxicity is paramount. Another class of hydrocarbons is naturally derived terpenes, including D-limonene [60]. This compound is the most preferred among other monocycloterpenes because of its low viscosity at low temperatures. It is obtained by distilling orange oil obtained from citrus peels [61,62], and is classified as a safe and environmentally harmless HTF, so it is usually used in the pharmaceutical and food industries. The solidification point of D-limonene is −78 °C, and below this temperature it has a dense gel-like form that cannot be pumped. Therefore, it is recommended to use this substance at a temperature no lower than −60 °C. Moreover, at temperatures above +50 °C, D-limonene oxidizes quickly in the presence of air, so it can be used in processes requiring temperatures below +50 °C [60].

### 2.5. Silicones

Silicones are another class of popular low-temperature heat transfer fluids. The most commonly used is polydimethylsiloxane, also referred to as silicone oil [9]. It is a synthetic polymer compound with molecular weight and thermophysical properties dependent on the length of the chain. The temperature range in which it is possible to use silicone oil is very wide, ranging from −100 to +260 °C [35,63]. This type of fluid is characterized by a long life in closed systems without oxygen access. Moreover, silicone oil is not very toxic and, with the use of appropriate additives, is also odorless, which makes it a non-burdensome medium for the personnel working with it. It exhibits a low surface tension, making it possible to leak at the joints of the wires, although thanks to this, it improves the wetting properties [64]. Silicones are typically more expensive than aromatic and aliphatic hydrocarbon-based fluids. Because of the negligible toxicity of this class of compounds, they are used mainly in the pharmaceutical industry.

### 2.6. Monohydroxyl Alcohols and Polyols

Monohydric alcohol aqueous solutions are also used as HTFs [65]. Methanol is a popular alcohol used in cooling mixtures due to its low cost and can be used down to -40 °C. Furthermore, it is possible to inhibit the corrosive properties of methanol solutions by adding appropriate substances. The main disadvantage of this solution is its high toxicity. It is classified as being more harmful than ethylene glycol, and therefore water solutions of methanol are used only in installations located outside the building. Moreover, methanol is a flammable liquid that poses a fire risk during use and storage. Ethanol is a less toxic alternative to methanol. However, it is also a flammable liquid, and its price is much higher than that of methanol. However, due to good thermal and transport properties (including low viscosity, nearly isentropic behavior, and not too low working pressure values), it is considered a working medium for organic Rankine cycle (ORC) technology [66]. On a micro-scale, it was successfully tested in a domestic ORC unit [67].

Alkylene glycols, most commonly ethylene and propylene glycol, are often used as components of liquid HTFs [68]. Ethylene glycol (EG) is practically odorless and miscible with water without restrictions [69]. Its aqueous solutions can be used at temperatures down to the −40 °C, although due to their high viscosity at low temperatures, they are most often used down to −10 °C. When used as a freezing point depressant, EG concentrations ranging from 30 to 60 vol% are commonly used [70]. Various types of additives are also applied to prevent corrosion, the formation of deposits, and foaming. Typically, they are used in concentrations of 0.1 to 3 wt% of EG, which also has very low vapor pressure compared to water. Because of the relatively low vapor pressure, mixtures of EG and water can retain their properties for longer periods compared to mixtures of water and more volatile alcohols. Although EG is effective as an agent to lower the freezing point and increase the boiling point of the coolant, its main disadvantage is its high toxicity. Therefore, it cannot be used in open systems and is not suitable for use in the food and pharmaceutical industries. Propylene glycol (PG) is used as a replacement for EG in many formulations of heat transfer fluid to avoid the toxicity associated with EG [71]. PG is so non-toxic that it has been approved as a food additive. The biggest obstacle to the more widespread use of PG as a base fluid in HTF concentrates is its relatively high cost compared to that of EG. Moreover, propylene glycol is more viscous than ethylene glycol. Therefore, similarly to EG, it is most commonly used down to -10 °C due to its high viscosity at low temperatures. It is also important that the addition of EG or PG reduces the heat transfer efficiency compared to pure water.

**Figure 4:**
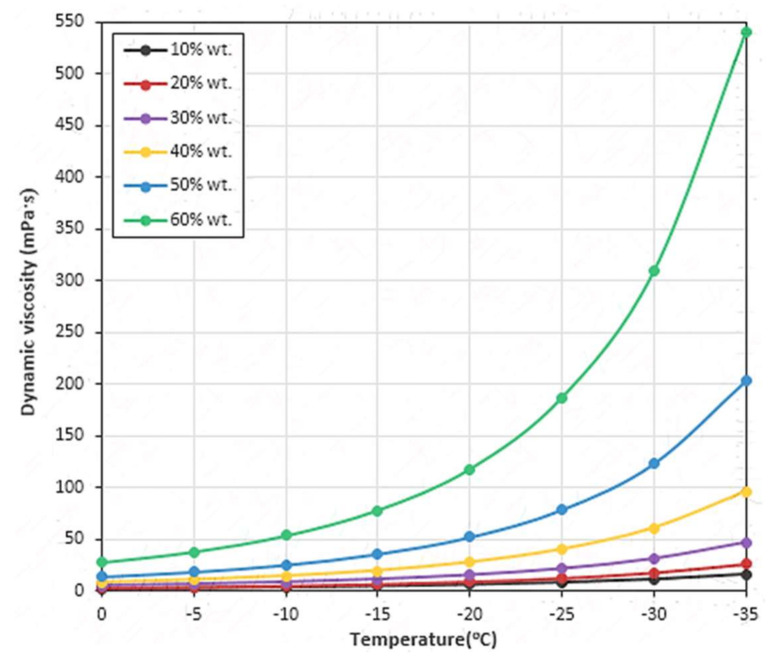
Dynamic viscosity of aqueous glycerol solution profiles versus temperature values depending on the mass concentration of glycerol, based on literature data [77].

An alternative to glycols is glycerol due to its non-toxicity and easy biodegradability [72]. Aqueous glycerol solutions are commonly used as heat transfer agents having indirect food contact during rapid freezing in the food industry [73]. However, glycerol and its aqueous solutions are characterized by high viscosity at low temperatures [74,75]. Figure 4 presents graphs of the relationships of dynamic viscosity of aqueous glycerol solutions on temperature (in the low range) and on the mass concentration of glycerol. Despite the fact that increasing the content of glycerol in the mixture with water causes a decrease in the freezing point, which results in an increase in the possible range of applications as an HTF, on the other hand, it causes a drastic increase in the solution viscosity, especially at the concentration value of 40 wt% and more, which is an undesirable phenomenon. The properties of glycerol–ethylene glycol–water mixtures were also investigated in terms of their use as HTFs. The solution that contained 40 wt% water and 60 wt% of glycerol–ethylene glycol mixture with a composition of 71.4 wt% glycol and 28.6 wt% of glycerol was characterized by the lowest pour point, which equaled −49.84 °C [76]. However, the greatest problem with such solutions is the high viscosity.

### 2.7. Summary of Environmental, Technical, and Economic Aspects of Traditional HTFs

Table 1 summarizes the types of heat and coolant transfer fluids, with a detailed comparison of the environmental, technical, and economic aspects of their application. The environmental aspects were evaluated based on the corrosivity and toxicity characteristics of the fluids. Thermal conductivity, rheological parameters, and Prandtl number were taken into account in assessing technical issues. Economic factors were compared considering the availability and price of the fluids. In the case of conventional HTFs, all of the media presented are highly available, which is their undoubted advantage.

When analyzing the data collected in Table 1, it is clear that the most important and greatest advantages of most traditional HTFs are low toxicity, high availability, and low price, as well as very good thermophysical properties in the temperature range in which they are applied. However, the biggest problem is their high corrosivity. Therefore, when looking for alternative HTFs, researchers should focus on solutions that have a low cost, low toxicity, and are readily available, have comparable or better thermophysical properties, and show significantly lower corrosivity towards the system’s construction materials.

## 3. Novel HTFs

### 3.1. Nanofluids

**Figure 5:**
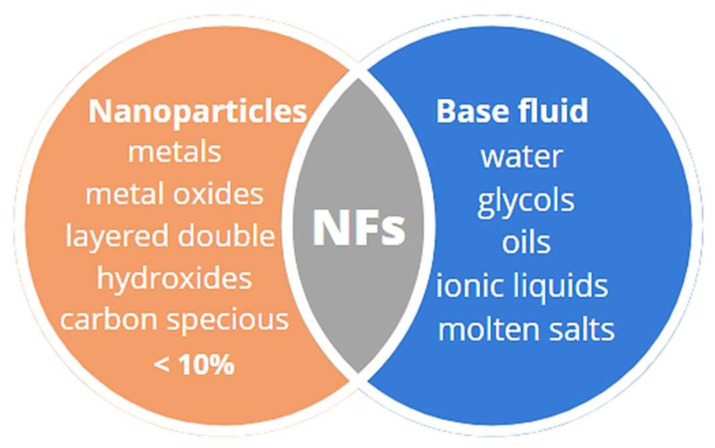
The composition of heat transfer nanofluids.

The first and vast group of novel transfer fluids is nanofluids (NFs) with a solid–liquid composition. In this system, nanoparticles (NPs) with an average size below 100 nm, for example, metals, metal oxides or hydroxides, and carbon compounds, are suspended in the base fluid such as water, oil, glycol or a water–glycol mixture, as is shown in Figure 5. An advantage of selecting water-based NFs is their non-toxic nature. Generally, the concentration of nanoparticles in the base fluid does not exceed 10%. The assumption of this technology is the incorporation of solid NPs that possess much higher thermal conductivity (TC) than the base fluid, which therefore can improve the heat transfer coefficient (HTC) [78,79]. Heat transfer improvement of nanofluids may be related to energy transfer by means of nanoparticle dispersion, Brownian motion of particles, particle migration, ballistic phonon motion, reduction in boundary layer thickness, and delay in the boundary layer’s development [27,80,81]. Iacobazzi et al. [82] investigated the effect of various mechanisms (layering, Brownian motion, clustering, ballistic phonon motion, thermal boundary resistance and mass difference scattering) on the thermal conductivity of NFs. For the first time, the authors suggested the examination of the effect of mass difference scattering. This mechanism was examined because it was the most intensive mechanism reducing the TC value of nanofluids, compared to that of microfluids. In the study, Milanese et al. [83] observed that layering phenomena could be different for metal and metal oxide NPs. In the presence of Cu NPs, two shell-like formations of water molecule layers were noticed, while for CuO, this phenomenon did not occur. This might explain the higher improvement of TC for Cu-based NF than for CuO NF.

In the application of nanometric particles, common problems limit the use of fluids with micrometric particle size—the clogging of small passages due to the large agglomeration of the solid fraction may be overcome. However, a crucial aspect is a proper alignment of the nanoparticles’ properties with the specific application requirements such as shape, size, thermal stability, chemical composition, and compatibility with the base fluid as well as content in the base fluid. Not only the properties of nanofluids, but also the geometry of heat exchangers may affect thermal performance [84]. For example, Visconti et al. [85] used a modified flat panel solar thermal collector to avoid sedimentation of the solid phase.

The stability of the NFs is one of the crucial requirements and challenges to be complied with in applications in thermal systems. The prepared suspension should have high stability over a long timeframe. However, the nanofluids may be unstable because of the strong van der Waals interactions and cohesive forces between the nanoparticles. Therefore, the use of appropriate preparation methods is key to its importance. To prevent the agglomeration of nanoparticles in suspension and improve the stability of nanofluids, different methods can be used, particularly pH control, surfactant incorporation, ultrasonication, surface functionalization or modification, or high-pressure homogenization [29,86,87,88]. Generally, the addition of the surfactants to the suspension is considered to be the most cost-effective method for obtaining a satisfactory stability of the composition. However, the addition of these substances may have a negative impact on the thermophysical characteristics of NFs [89]. Taking into account the stability, the content of the nanoparticles must be kept at an optimal level. A growth of NPs content causes a higher density of NPs per unit volume. This leads to more frequent particle collisions and a higher possibility of clustering [90]. Typically, the idea is to achieve an improvement in the thermal characteristic but not to increase the fluid viscosity, and hence pumping power requirements along with cost efficiency. To evaluate the stability of NFs, methods such as zeta potential measurement, absorbance spectrometry, and light scattering measurements are commonly implemented [91].

#### 3.1.1. Metal Oxides

The first subgroup of nanofluids includes metal oxide nanoparticles. Among them, the most frequently reported are Al_2_O_3_, CuO, ZnO, SiO_2_, and TiO_2_ [92,93]. Researchers widely implemented these materials because of their relatively low cost and easier production than other NPs. This is beneficial, especially in large-scale production. The advantages of metal oxide NPs compared to metal NPs include resistance to oxidation and chemical stability, and good thermal properties. In addition, several metal oxides have a lower density than metals, which prevent sedimentation when used for nanofluid preparation [93,94,95].

The addition of metal oxide NPs led to an increase in the heat transfer coefficient compared to the base fluid. Several studies have found that a higher concentration of metal oxide NPs causes a higher thermal conductivity, an increase in Brownian motion, and thermophoresis effects [96]. Thus, the heat transfer coefficient (HTC) increases with the incorporated metal oxide NPs. This suggests that when the NP content in the fluid increases, a greater proportion of its atoms are near the surface, and the surface of the NPs effectively participates in the heat transfer. Moreover, among the factors that affect the heat transfer, the researchers pointed out the synergistic effect of the interaction between metal oxide NPs and fluid, increasing the turbulent flow and improving the cross-sectional gradient of fluid temperature [97]. However, it is important to note that when the optimal content of NPs of metal oxide in the base fluid is exceeded, the HTC value may decrease.

The increase in HTC with the application of NFs instead of the base fluid is correlated with an increase in the effectiveness of the heat exchanger, which led to the reduction in the heat exchanger size to obtain the same heat exchange performance and therefore reduce energy consumption and total costs [98]. Pourfattah et al. [99] observed that the addition of 4 vol% of Al_2_O_3_ at a Reynolds number of 60,000 causes an augmentation of heat transfer to 22% as compared to pure water. Manetti et al. [100] concluded that the incorporation of Al_2_O_3_/water NFs led to a decrease in wall superheating of up to 32% and 12% for the smooth and rough surfaces, respectively, compared to pure water, which consequently leads to an improvement in thermal systems’ efficiency and safety. Meanwhile, Mansoury et al. [101] compared the effect of nanofluid application in various types of heat exchangers. The authors observed the highest enhancement for the double-pipe heat exchanger (DPHE) with 1 vol% Al_2_O_3_. A 60% growth in the Nusselt number was observed in comparison with water, while that for the plate HE was not significant. Yasinskiy et al. [102] investigated the thermal properties of TiO_2_ nanoparticles suspended in an oil-based mixture of diphenyl oxide and biphenyl with 1-octadecanethiol as a heat transfer fluid in CSP. For 2.44 vol% of the nanoparticles, the thermal characteristics were significantly enhanced, up to 52.7% for the isobaric specific heat, and up to 25.8% for the TC. Salimi-Yasar et al. [103] described the application of an oil-based TiO_2_ nanofluid in a drilling processes as a cutting fluid. All prepared nanofluids had higher Nu than the base fluid, while the maximum enhancement was 23.7% for 1 wt% and a Re of 900. The researchers concluded that the application of the oil-based TiO_2_ nanofluid decreased the operational temperature of the drilling process and improved the heat transfer rate. Ahmed et al. [104] claimed that the application of ZnO/water NFs in a circular tube heat exchanger led to an improvement in HT of 50% (1000 to 1500 W/(m^2^·K) with the Re from 5849 to 24,544) and a 49% growth in Nu number for 0.1 wt% of ZnO compared to pure water.

Recent evidence reveals that the use of metal oxide NFs with improved thermal characteristics as a coolant instead of conventional fluids makes it possible to design compact size radiators, and reduce drag and save fuel cost [81,105]. Goudarzi et al. [106] used Al_2_O_3_/EG as a cooling transfer fluid in the car radiator. The authors concluded that the Nusselt number and the friction factor for nanofluids were higher than those for pure EG. Eid et al. [107] investigated the heat transfer characteristics of large-surface TiO_2_/EG nanofluid for coolant applications. An improvement in TC of 8.7% was observed for the application of 0.2 vol% TiO_2_. According to experimental and numerical calculations, 0.2% TiO_2_ increased the coolant surface heat transfer rate by approximately 24% compared to pure EG, which could be due to the higher TC value. Krishnakumar et al. [108] prepared the ethylene glycol/water (60:40) nanofluid with the addition of TiO_2_ nanoparticles. The maximal augmentation in TC was approximately 24% at 0.8 vol%, while the viscosity increased by approximately 16%. For convection measurements, the greatest enhancement in HTC was 116% with a Re number of 2050 and 0.5 vol%. Islam et al. [109] characterized the TC of TiO_2_/water-EG(50:50) nanofluid as a coolant for proton exchange membrane fuel cells. The authors noticed that the addition of 0.5 vol% TiO_2_ NPs increased TC by more than 10% compared to the base fluid. Furthermore, Khan et al. implemented ZnO/water-EG(50:50) as a coolant in a car radiator. With an increasing nanofluid flow rate, HTC also increased. A maximum improvement in heat transfer of up to 36% was recorded for 0.04 vol% of ZnO. A higher heat transfer rate was observed at a low flow rate compared to a higher flow rate. However, an extra 2.5% pumping power was needed for the radiator using 0.04% ZnO NP with water-EG(50:50) coolant compared to the base fluid in the same radiator. Meanwhile, Colangelo et al. [110] described the dynamic model to evaluate the efficiency of the heating ventilation air conditioning system working with a water–glycol mixture or an Al_2_O_3_ nanofluid. The model was experimentally validated based on a real plant. The results confirm that the application of nanofluid increases the efficiency by about 10% and reduces the electrical energy consumption of the system.

An important parameter characterizing metal oxide NFs is the stability of the NP suspensions. High stability and low tendency should describe the prepared nanofluids regarding agglomeration and consequently sedimentation. Iacobazzi et al. [111] characterized the effect of clustering of nanoparticles on thermal conductivity. It was observed that small clusters decreased the TC, while large clusters improved the TC. This phenomenon could be related to NP sedimentation, which causes convective motion generated by particle movement within the fluid under the effect of gravitational force. However, measurements of zeta potential and backscattered light demonstrated that clustering reduces the thermal conductivity of the nanofluid.

In the research performed by Said et al. [98], CuO/water nanofluid was used in the shell-and-tube heat exchanger. As prepared, the nanofluids were characterized by a zeta potential greater than ±30 mV, suggesting that they could maintain their suspension stability. The stability of the suspension depends on the compatibility of its components. Javed et al. [112] recommended the use of a CuO/waste palm oil nanofluid for heat transfer application. Analysis confirmed that the prepared nanofluids showed excellent stability for at least 6 months with the highest improvement in TC with 0.7 wt.% of CuO (enhancement of up to 190% compared to palm oil waste). In some cases, the appropriate solution is the addition of stabilizers to prevent suspension instability. Khanlari et al. [86] applied a water-based TiO_2_ nanofluid in the plate-type heat exchanger. The TiO_2_ concentration (size about 14 nm) equaling 2 wt.% increased the heat transfer coefficient to about 6%. Authors concluded that the addition of Triton X-100 surfactant (0.2 wt.%) prevents sedimentation and flocculation issues, as well as accumulation inside the plate heat exchanger. Another possibility is the idea proposed by Ilyas et al. [88], who analyzed the behavior, thermal, and rheological properties of functionalized ZnO-paraffin oil nanofluids. The ZnO nanoparticles were functionalized with oleic acid to improve the stability of the suspensions. The authors noticed that the modification of ZnO was essential to improve the stability of oil-based nanofluids. f-ZnO NFs had a much smaller average particle size than the unmodified ZnO NPs. The highest enhancement in the effective viscosity was about 17% for 1 wt% f-ZnO-oil NF at 55 °C. The coefficient of thermal expansion was found to be reduced with increasing f-ZnO content.

However, the increases in the viscosity, friction factor, and thus pressure drop have become important issues associated with the incorporation of metal oxide NPs. The growth of these parameters is related to higher pumping power requirements and consequently leads to an increase in process cost. Gkountas et al. [113] concluded that for 5% nanoparticle loading, HTC improvement reached 75%, while the pressure drop increased up to 8%, compared to pure water. Du et al. [114] investigated the application of CuO/water nanofluid as a heat transfer fluid in geothermal heat exchanger (GHE). The results presented confirm that the incorporation of nanofluid may increase the pumping power consumption of GHE by 16.6%. The ratio between the heat load and the pumping power of the NF increases by 20.2%. Zhong et al. [115] analyzed the heat transfer characteristics of highly self-dispersed TiO_2_ nanofluid in the multiport minichannel. The addition of 1 vol% of nanoparticles increased fluid viscosity by about 14.9% and caused larger friction factors (up to 41.6%). Moreover, Wen et al. [116] observed that the addition of ZnO NPs to water resulted in a remarkable increase in viscosity. In addition, the authors noticed that the pressure drop and fanning friction factor of nanofluid were notably higher than those for pure water and increased with nanofluid content. The friction factor increased by 40.9% for 1.5 vol%, which was mainly caused by the enlarging of the viscosity. However, the thermal performance factor of the ZnO nanofluid was maintained at about 1, suggesting that the advantage of heat transfer improvement compensated for the limitation induced by the increase in pressure drop.

The thermal characteristics of metal oxide NFs may also depend on the shape, size, or crystallic form of NPs. Shahrestani et al. [117] noticed that an increase in the volume fraction of nanoparticles may increase the heat transfer coefficient. However, the increase in the diameter of the nanoparticles may decrease the heat transfer coefficient. Authors noticed that an increase in the nanoparticle radius results in a reduced effective thermal conductivity. Innovative research was presented by Zhu et al. [118], who compared the use of CuO nanospheres and nanowires suspended in water. The diameter of the particles was varied from 30 to 80 nm, while the length was varied from 3.5 to 5.5 µm, respectively. CuO nanowires containing nanofluids have a thermal conductivity that is higher than that with nanospheres. The maximum increace in the thermal conductivity of the nanofluid with CuO nanowires reached up to 60.78%. Ahmadi et al. [119] modeled the heat transfer properties of CuO suspended in ethylene glycol. Based on the presented results for the analyzed data, it can be concluded that the highest influence on the thermal conductivity ratio of CuO/EG fluid is the temperature, followed by the size of the nanoparticles. The least significant parameter was the concentration of CuO. Esfe et al. [120] evaluated the influence of the size of TiO_2_ nanoparticles (10–50 nm) suspended in water on the convective heat transfer and pressure drop. The highest increase in Nu number was observed for nanoparticles with an average size of about 20 nm and a concentration of 1.5 vol%. With a higher TiO_2_ content, a greater pressure drop is seen in the nanofluid. However, when the particle size is reduced, it decreases the pressure drop. This may be explained because in a volume fraction, when the size of the nanoparticles decreases, the surface area increases, while in turn the velocity gradient in the fluid has more variation and therefore the pressure drop will increase. The effect of the crystal form of TiO_2_ in the nanofluid on the heat transfer characteristics in the microchannel for TES was evaluated by Ding et al. [121]. Rutile and anatase forms of TiO_2_ were compared. The authors found that the rutile–water nanofluid had better stability than the anatase–water nanofluid, and the TC value was higher than for the anatase–water system, with a maximal increase of 3.27% in comparison with pure water. The viscosity of rutile-water increased by about 4.87%, while that of anatase-water by about 7.45%. For 1 wt% of the rutile–water nanofluid, Nu increased from 6% to 41%, while the increase in the pressure drop was maximally 8%.

Milanese et al. [122] investigated the optical behavior of various water-based nanofluids applied in solar power systems. In all experiments, transmittance grew, passing from the visible to the infrared region. It was noted that the application of 0.05 vol% TiO_2_, which can completely absorb solar radiation at a depth of 1 cm, led to the minimizing of the pumping and maintenance costs. Moreover, the authors characterized the possibility of application of NPs in the gas-based system [123]. It was observed that the optical properties of ZnO, Fe_2_O_3_ and CeO_2_ gas-phase NFs were not affected by the growth in the temperature in the range 25–500 °C. These results confirm that such NPs can operate at very high temperatures. An interesting direction is a solution developed by Potenza et al. [124] with the application of an air-based CuO NP working fluid in a parabolic trough collector. The authors pointed out the deposition of NPs within the receiver pipe due to humidity as a critical issue related to the application of this type of HTF.

#### 3.1.2. Metals

The next subgroup of nanofluids includes the material based on the addition of metal nanoparticles such as Cu, Al, Fe, Ni, Si, Ag, etc., to the base fluid [125]. Metallic species are applied because of their high thermal conductivity coefficient, significantly higher in comparison with their oxides. Nakhchi and Esfahani [126] investigated the possibility of applying a Cu/water nanofluid in the heat exchanger tube equipped with cross-cut twisted tape. The authors concluded that thermal performance was enhanced by increasing the content of NP inside the duct, increasing up to 46% for 1.5 vol% compared to the base fluid. The results show that the HTC increased up to 23.20% with the increasing NP content from 0 to 1.5 vol%. The researchers explained this fact by the fact that the higher Re improves the greater swirling motion, causing a smaller thermal boundary layer thickness along the channel wall, while collisions between nanoparticles and between nanoparticles and the tube wall lead to higher energy exchange. However, the friction factor of the 0.1-1.5 vol% nanofluids was up to 1.33 times higher than that of pure water. Mebarek-Oudina and Bessaïh [127] performed a numerical analysis of natural convection heat transfer of a Cu–water nanofluid in a vertical cylindrical annulus. The researchers observed that with the increase in the NP concentration, the maximum temperature decreases and the heat transfer increases. For the Ra value of 10^4^, the average Nu number grew with the increase in the NP content. This was related to the enhancement of the effective TC of the nanofluid as the number of NPs increased. Gholamalipour et al. [128] investigated the usage of a Cu/water nanofluid inside a porous annulus. In all the Ra and Da numbers, when the NP content increased, the average Nu number increased and reached the highest value at 4 vol%. Furthermore, at Ra = 104, the highest effect of nanoparticle addition on enhancing the heat transfer was up to 13%. The positive impact of NP addition on the heat transfer was explicable because the stream functions were compressed near the walls, which led to the velocity increment and improvement of the heat convection process. Saleem et al. [129] compared the heat transfer characteristics of different forms of copper NP (spherical, blades, and platelets) suspended in a water-based nanofluid. The velocity of a fluid drops by aggregation of the solid fraction. The authors noticed that platelet-shaped nanoparticles offered the greatest improvement in heat transfer, providing the maximum increase in velocity, viscosity, and temperature compared to the NFs based on spherical and blade-shaped nanoparticles. Hadavand et al. [130] studied the coolant properties of a Ag/water nanofluid in a semi-circular lid-driven cavity. The presence of Ag nanoparticles at higher concentrations (6 vol%) in the nanofluid, with the higher TC and enhancement of the Nu number, leads to better temperature distribution with smaller temperature gradients, and thus improved cooling performance of the cavity. Mir et al. [131] investigated the heat transfer performance of Ag/water nanofluid in an elliptical curved minichannel. The researchers observed that an increase in the number of NPs leads to an increase in the temperature of the central line of flow and an increase in Nu number. The presence of Ag NP in the nanofluid induced the destruction and postponement of the creation of the thermal boundary layer as well as the extension of the collision of NPs with each other and heated surfaces. Furthermore, the higher content of NPs leads to the growth of the local friction factor, which is related to the rise in the viscosity and density of the fluid and, as a result, to the augmentation of shear stress and fluid momentum depreciation. Saleh and Sundar [132] evaluated the heat transfer characteristics of Ni/water NFs in a corrugated plate heat exchanger. At 0.6 vol% of NPs and 60 °C, the TC and viscosity increased by approximately 33.92% and 67.45%, respectively, compared to pure water. The addition of nanoparticles augmented the overall HTC, as well as the Nu number, by up to 38.60% and 42.68% (for 0.6 vol%), while the thermal entropy generation was reduced by 15.70%. However, frictional entropy generation and pumping power increased by 68.29% and 61.77%. Esfe et al. [133] investigated the thermal conductivity and viscosity of Fe/water nanofluids with different sizes of NPs (37, 71, and 98 nm). TC increased as the NP content increased, while the NP size decreased. However, the dynamic viscosity of NF was enhanced with higher concentration and diameter of NPs. Khoshvaght-Aliabadi et al. [134] compared the performance of Ag/water, Fe/water, and Cu/water NFs in a U-tube heat exchanger with a concentration of 0.1 vol% NP. The authors observed that the HTC and pressure drop of all metallic nanofluids were higher than those of pure water. Furthermore, the highest values were recorded for Ag/water NFs, with the highest augmentations of 18.2% and 8.5% for HTC and pressure drop, respectively. Research on nanofluids with metal nanoparticles is developing to utilize their magnetic properties and a strong magnetic field to obtain a higher level of heat transfer enhancement. Numerical studies on the application of an external magnetic field to nanofluids have been reported, considering various concentrations of nanoparticles: copper [135] and silver [136] were reported. The experimental analyses are less common, but they are presented in [137] and [138]. From the numerical analysis, there is a conclusion that the strong magnetic field attenuates the convection, so its negative influence was observed. An opposite effect was observed in the experimental studies; the enhancement of heat transfer processes caused by a strong magnetic field was exhibited. The most probable reason for such different results comes from the method of nanofluid treatment, such as using a single-phase medium (typical numerical approach) or a two-phase one.

#### 3.1.3. Layered Double Hydroxide (LDH)

One more subgroup is layered double hydroxides (LDH). Generally, this is the class of two-dimensional layered anionic structures. Their significant advantages include their flexible and tunable chemical composition, as well as their high chemical and thermal stability [139]. These compounds mainly include Fe, Al, Ni, Zn, and Cu hydroxides. Chakraborty et al. [140] investigated the potential application of Cu-Al LDH nanofluid as a coolant in a pressure atomized spray. The authors evaluated the effect of the Cu:Al molar ratio on TC, stability, and heat transfer characteristics. All suspensions showed zeta potential values above ±30 mV, thus being classified as highly stable. The optimal ratio of Cu:Al was 4:1. NF with 0.016% NPs achieved the highest improvement in TC with a 15.17% increase compared to pure water. However, the viscosity values remarkably increased with a higher NP loading, at 0.024% reaching 57.16% increase. In another study, Tiara et al. [141] compared the influence of various surfactant additives with Cu-Al LDH applied as a heat transfer fluid in jet impingement on a hot steel surface. The nanofluid with Tween 20 showed the highest TC improvement and the maximal decrease in surface tension. In addition, viscosity was significantly reduced upon incorporation of Tween 20 surfactant. This nanofluid reached the highest cooling rate of 154 °C/s and the critical heat flux (CHF) value of 3.06 MW/m^2^. Chakraborty et al. [142] applied ternary LDH compounds such as Cu-Zn-Al(4:1:1) in spray cooling. In this case, the highest TC (0.68 W/(m·K)) was obtained for 0.016% of LDH and was about 13.9% higher than pure water. Meanwhile, for 0.024% the viscosity of the nanofluid was 68.7% higher than for water. The maximum cooling rate was achieved for 0.016% and was 158 °C/s (18.5% higher than pure water), thus suggesting that this value was significantly higher than above the ultra-fast cooling range (>133.3 °C/sec for a plate of 6 mm). At 0.016% of the LDH content, a 15.6% reduction in coolant consumption compared to water was achieved. Furthermore, the average HTC value of 2340 W/(m^2^·K) was recorded for 0.016% of Cu-Zn-Al LDH nanoparticles.

#### 3.1.4. Carbon Species

Researchers commonly introduce carbon species into the base fluid to improve the heat transfer characteristics. Among them, graphene, graphene oxide, carbides, MXenes, carbon nanotubes, and many more may be distinguished.

The SiC nanofluid is characterized by relatively low cost, stability, widespread availability, and simple preparation processes [143]. Ponnada et al. [143] applied the SiC/water nanofluid in a circular tube and investigated the effect of volume concentration and NP size. The authors concluded that heat transfer grew with increasing NP content and NP size. The HT augmentation was up to 36.74% compared to the base fluid. Moreover, the friction factor increased with a higher amount and size of NP. Al-Waeli et al. [144] characterized the application of SiC/water nanofluid in a photovoltaic thermal system. The authors observed that the addition of 3 wt% of SiC NPs increased NF density by up to 0.0082% and viscosity by up to 1.8%, compared to water. Meanwhile, TC was improved by 8.2% and thermal efficiency increased by 100.19%. The suspension showed high stability and usefulness over a long timeframe; after three months, TC was only reduced by 0.003 W/(m·K). Srinivasan et al. [145] compared the thermal characteristics of various base oils (engine, furnace and transformer oils) with the addition of SiC nanoparticles. For all base oils, the authors observed the highest enhancement of heat capacity for 2 vol% of SiC. Moreover, the SiC/engine oil nanofluid showed the highest growth in heat capacity by up to 8.97 times compared to the base fluid.

Important examples of nanofluids also incorporate carbon nanotubes (CNTs). The thermal conductivity of CNTs is variable and depends on the length of the particles and their type (SWCNT and MWCNT). MWCNTs possess very high thermal conductivity, up to 3000 W/(m·K) at room temperature [146]. Moradi et al. [147] characterized the flow and heat transfer properties of MWCNT/water nanofluid. The authors observed the highest HTC growth (about 35%) for the lowest MWCNT NP concentration—0.04 vol%. Sarafraz et al. [148] investigated the fouling formation and thermal properties of CNT/water nanofluids in a heat sink. The nanofluids presented a higher HTC and lower temperature profile inside the heat sink compared to pure water. The increase in NP mass concentration improved the HTC to a large extent. Fouling thermal resistance is strongly dependent on the NP content, and at a higher NP amount, a shorter operating time is required to reach the constant fouling thermal resistance. Fan et al. [149] characterized the impact of the length and diameter of the MWCNT on the heat transfer performance. The length of the CNT was 1 or 5 µm, while the diameter was about 30 or 60 nm. The high stability of the samples was observed after four weeks. Critical heat flux grew by about 60%. The authors assumed that CNTs with a length of 5 µm and a diameter of 60 nm presented the highest enhancement in quenching and boiling behaviors. Abdeen et al. [150] evaluated the influence of the carbon nanotube content in the CNT/water nanofluid on the corrosion performance. The CNT nanofluids showed relatively low corrosion rates, comparable to those of tap water and deionized water. The lowest corrosion rate was observed for 0.1 wt% CNT nanofluids at room temperature. The inhibitory properties of CNTs at low concentrations were related to the physical adsorption of CNTs on the surface of the metal and the formation of a barrier to prevent attack of the metal’s surface by the solution ions. However, the inhibition efficiency decreased with increasing CNT concentrations.

Additionally, graphene and graphene oxide NFs were used as a heat transfer fluid. Graphene materials have significant advantages such as high flexibility and thermal conductivity, chemical inertness, and high density [151,152]. The heat transfer characteristics of the graphene nanoplatelet fluid in a microchannel were investigated by Sarafraz et al. [80]. The higher content of NP led to an increase in TC, heat capacity, and density, with the highest improvement being by 27%, 16.8%, and 5%, respectively. This heat transfer enhancement was correlated with the thermophoresis effect. The movement of graphene towards the cold environment was related to the temperature gradient and the density variation between the hot and cold regions. Graphene NPs absorb the thermal energy as sensible heat and transport it to the cold environment, causing the heat transfer from the hot surface towards the working fluid. Sözen et al. [153] used a graphene/water NF with 2 wt% of NPs to improve the thermal performance of air-to-air heat recovery units. The authors concluded that the graphene/water NF was more efficient than pure water, with a maximum thermal efficiency value of 34.1%. The maximum rate of improvement in thermal efficiency was 87.7%. An application of nanofluid minimized the thermal resistance of the heat pipes; thus, the HT grew. The highest reduction in thermal resistance was 52.3%. The use of nanofluid with graphene NPs provided the difference between the average evaporator and condenser temperatures of the heat pipe to reach lower values. Consequently, boiling occurred at lower temperatures. Selvam et al. [151,154] used a graphene/water-EG(70:30) nanofluid as a coolant for the automobile radiator. The authors observed that addition of graphene NP led to an increase in the convective heat transfer coefficient (up to 51%) and the overall heat transfer coefficient (up to 104%). Meanwhile, the pressure drop increased with the NP loading.

A novel class of 2D carbon compounds used as a heat transfer fluid is the MXenes. A major example of these is titanium carbide (Ti_3_C_2_T_x_). Ti_3_C_2_T_x_ possesses metallic-like and ceramic properties with high thermal and electrical conductivity, and resistance to heat [155]. Bao et al. [156] characterized the TC, stability and viscosity of Ti_3_C_2_T_x_/EG. The researchers observed that the nanofluid with 1 vol% delaminated Ti_3_C_2_T_x_ presented high stability within 30 days. The TC of NF with 5 vol.% of delaminated Ti_3_C_2_T_x_ increased by 64.9% compared to pure EG. MXene NFs presented Newtonian fluid properties. Aslfattahi et al. [157] investigated the potential of MXene/silicon oil nanofluid application as a heat transfer fluid in the concentrated photovoltaic thermal collector. The 0.1 wt% MXene/silicon oil nanofluid was found to be thermally stable up to 380 °C. The highest TC improvement of about 64% was observed for 0.1 wt% MXene/silicon oil compared to base silicone oil at 150 °C. The viscosity of silicone oil with the addition of Mxene NPs was independent of the content of MXene nanoparticle and was reduced by 32% when the temperature increased from 25 to 50 °C. Viscosity reduction is a desirable requirement, as this leads to reducing the pumping power in a flow channel. Samylingam et al. [158] used a composition of MXene/palm oil as a heat transfer fluid in the PV/T system. For 0.2 wt%, MXene/palm oil showed a 68.5% higher TC than pure palm oil at 25 °C, while viscosity reduction reached 61% when the temperature increased from 25 to 50 °C. The unique feature of MXene NF was the negligible increase in its viscosity with an increase in the NP content. Similar research was performed by Rubbi et al. [159] who applied MXene/soybean oil in the PV/T system. The zeta potential values revealed the physical stability of the NF. The TC of Ti_3_C_2_T_x_/soybean oil for 0.125 wt% of Ti_3_C_2_T_x_ showed an improvement of 60.82% at 55 °C, while the specific heat increased by about 24.5% compared to pure soybean oil. Moreover, the surface temperature decreased by 14 °C by applying the NF as a cooling fluid. Both studies showed that these NFs had better thermal performance than Al_2_O_3_/water composition. Samylingam et al. [158] showed that MXene/silicon oil NFs reached an increase in thermal efficiency of about 16%, a flow rate of 0.07 kg/s, and a growth of HTC of about 9% compared to the Al_2_O_3_-water heat transfer fluid. Meanwhile, Rubbi et al. [159] reached the highest increase of 14.3% at 0.06 kg/s for MXene/soybean than for alumina/water NF.

#### 3.1.5. Hybrid Nanofluids

The application of hybrid nanofluids is one of the solutions to improve the thermal characteristics. Hybrid nanofluids are compositions with at least two different nanoparticles suspended in the base fluid. This method allows higher efficiency to be obtained because of the synergistic effect of NPs on the heat transfer properties. These hybrid nanofluids include, in particular, the combinations of metal–metal, metal oxide–metal oxide, and metal–metal oxide nanoparticles, as well as metal oxide–carbon compounds.

Hassan et al. [160] combined Cu and Ag nanoparticles (50:50 *v*/*v*) in water and compared the, with a single NF with Cu and Ag, respectively. The temperature profile of the hybrid nanofluid was improved compared with that of the Cu/water nanofluid because of its high heat capacity enhancement by the addition of Ag NP. The highest wall shear stress was observed at the wall for Ag/water, while it was reduced when a hybrid nanofluid was prepared. It was concluded that a hybrid NF may develop heat and mass flow and HT rate. Akram et al. [161] performed a theoretical investigation and compared the properties of Ag/water NF with hybrid Ag/Au-water NF. The velocity of the hybrid nanofluid was lower than that of the Ag/water nanofluid. With the addition of Ag NPs to water, an increase of up to 31% in TC was noticed, while when an equal amount of gold nanoparticles was incorporated into the silver–water nanofluid, the TC increased to 71.53%.

Hayat and Nadeem [162] compared the thermal properties of CuO/water NF and hybrid Ag/CuO/water NF. The application of hybrid nanofluids (Ag–CuO/water) led to a further increase in the heat transfer rate. Ag–CuO/water NF reduced fluid velocity and reached a higher temperature than a single nanofluid (CuO–water). The reason was that more massive particles overcome the single NP fluid flow. A major conclusion based on the performed research was that hybridity promotes the temperature distribution and the HT rate at the surface. Ghadikolaei et al. [163] investigated the potential of the combination of TiO_2_ and Cu NPs in the water-based nanofluid. The authors evaluated the influence of the NPs shape (brick, cylinders, and platelets) on the thermal properties. The highest temperature occurred for platelet NPs and hybrid TiO_2_-Cu/water nanofluid, compared to Cu/water NF, and other NPs shapes. The local skin friction coefficient for hybrid nanofluids was lower than that for nanofluids, whereas the value of Nu number for hybrid nanofluids was higher than that for the single nanofluid.

Gürbüz et al. [164] studied the application of hybrid CuO/Al_2_O_3_/water NFs in a U-type tubular heat exchanger. The use of hybrid nanofluid led to higher growth in overall HTC compared to CuO/water NF. The maximum enhancement in HTC of the hot side applying 1 wt% of CuO/Al_2_O_3_ NPs achieved 12.9%, while the highest increase in HTC of the cold side was 11.5%. Minea [165] compared the heat transfer characteristics of Al_2_O_3_/water, Al_2_O_3_/SiO_2_/water, and Al_2_O_3_/TiO_2_/water NFs. The highest HT enhancement was observed for the composition containing 2.5% Al_2_O_3_ and 1.5% of SiO_2_ (enhanced by 257 and a maximum enhancement of 241% in the Nusselt number. This was related to the highest viscosity and good TC. However, the application of these NFs caused higher pumping power requirements.

Gupta et al. [166] studied the potential of hybrid metal/COOH-functionalized MWCNT/water NFs. Metal NPs were Cu, Zn, Fe, and Ag, respectively. Among all the measured hybrid metal/COOH-MWCNT NFs, Cu/COOH-functionalized MWCNT/water presented the highest stability and remained stable for more than 45 days without the addition of any stabilizer. This composition had the highest TC with respect to water, with an enhancement of 78.5%. This was caused by the covalent attachment of Cu NPs to MWCNT, which promotes strong coupling. In the case of hybrid Cu/COOH-MWCNT NFs, the viscosity did not change significantly with the shear rate; thus, the pumping power would remain constant.

Kumar and Sarkar [167] applied 0.01 vol% Al_2_O_3_-MWCNT/water nanofluid in a minichannel heat sink. The highest improvement in convective HTC of 44.02% was observed with MWCNT nanofluid compared to the base fluid. However, the highest pressure drop, increasing by 51.2% as compared to the base fluid, was recorded for the MWCNT NF. The performance evaluation criteria had a value >1 for all nanofluids, suggesting that nanofluids were a more effective coolant compared to pure water, with the greatest value being 1.26 for MWCNT NF. However, the optimal mixing ratio of Al_2_O_3_ and MWCNT was 3:2, for which the hybrid nanofluid had the highest HTC to pressure drop ratio.

Hussien et al. [168] evaluated the potential application of a hybrid MWCNT/graphene NPs nanofluid in a mini-tube. Hybrid MWCNT/GNP nanofluids reduced the wall temperature in comparison with pure water and MWCNT nanofluids. The introduction of GNPs into MWCNT/water nanofluids caused an average improvement in HTC of 8.8%. The high ability of hybrid NF to transfer heat was related to the developed contact surface area and improved TC. Maximal enhancement occurred for the hybrid 0.25 MWCNT/0.035 GNP nanofluids at Re = 200 with an increase of 43.4% and a 11% increase in pressure drop. Meanwhile, Said et al. [169] studied the thermal characteristics of hybrid functionalized carbon nanofibers/reduced graphene oxide NFs. The NPs content was 0.04 vol% and the hybrid NF presented excellent stability within 6 months. The TC values were highest for the hybrid nanofluids and a maximum thermal conductivity of 0.798 W/(m·K) was observed at 55 °C. The viscosity of hybrid NFs was lower than that for rGO/water and functionalized carbon nanofibers/water monofluids.

### 3.2. Supercritical CO_2_

The second example of novel HTFs is supercritical carbon dioxide (s-CO_2_), due to its highly desirable non-flammable, non-explosive, odorless, non-toxic, and non-corrosive nature [170]. Moreover, it is readily available, inexpensive, and stable at high temperature. The operational temperature range is -73 °C to 1000 °C [171]. Carbon dioxide acts as a supercritical fluid above its critical temperature (31.1 °C) and critical pressure (7.38 MPa). This state, when CO_2_ adopts intermediate properties between liquid and gas (gas with a density like liquid), may easily be reached. An outstanding capacity to withstand very high temperature and excellent thermal features characterize s-CO_2_, and thus it may be an effective HTF. It is important that in the near-critical region, the density of s-CO_2_ for variable pressure does not show substantial growth. The thermo-physical characteristics of s-CO_2_ are temperature-dependent. Notably, near the pseudocritical temperature, the specific heat approaches its highest value. This shows differences in flow and HT characteristics compared to conventional fluids. The TC of s-CO_2_ increased with the bulk temperature in the subcritical region, whereas near the critical point, the TC decreases first and approaches the lowest value, and then grows to the highest value before lowering to a flat line. A significant drawback is that near the critical region, there occurs a rapid change in the properties of s-CO_2_. When the temperature distribution of the s-CO_2_ flow reaches pseudocritical temperature, the notable density gradient at a pressure slightly above a critical value (7.6 MPa) generates buoyancy effects [170]. Buoyancy has a prominent influence on heat transfer due to the deformation of the flow velocity profile. Consequently, the heat transfer coefficient decreases. Wang et al. [170] concluded that the buoyancy effects are greater with higher heat flux values. Secondary circulation becomes more visible at higher heat flux levels and increases the temperature variation between the top and bottom tube surfaces. Wang et al. [172] and Zhang et al.[173] confirmed that HTC in the horizontal tube are higher than in the vertical tube, because the disturbance of secondary flow in the horizontal tube overcame the limitations of temperature and velocity boundary layers on convection HT. Many researchers investigated the benefits and limitations of various applications of s-CO_2_, such as HTFs. s-CO_2_ has a high potential to eliminate heat exchangers in solar plants, because it can be used simultaneously as an HTF for the solar collector and as the working fluid for the power cycle. This resulted in more efficient and less complex power units [171]. Wang et al. [174] observed that in parabolic trough solar receivers, the non-uniform solar flux distribution can induce significant secondary flow of s-CO_2_, which improves the synergy of the velocity vector and the temperature gradient in the fluid, benefiting the convective heat transfer. Meanwhile, Wang et al. [172] observed that in a helically coiled tube, with increasing heat flux, the HTC decreased in the pseudocritical temperature region, whereas the HTC was almost unchangeable in the liquid-like region. While Guo et al. [175] noticed that in a mini tube, HTC decreased with increasing heat flux and decreasing mass flux, but was minimally affected by pressure because the wall temperature was away from the pseudocritical point. Moreover, under a high heat flux/mass flux ratio, the buoyancy effect was important and reduced the HT. It is an important feature that geothermal systems using CO_2_ as the working fluid achieve CO_2_ sequestration in deep reservoirs due to the fluid loss effect. In their research, Zhang et al. [176] described the comparison of supercritical CO_2_ used in an artificial smooth parallel-plate fracture and a rough and tortuous fracture that mimics rock fracture in geothermal systems. Compared to the smooth parallel-plate fracture, the CO_2_ flowing through the rough and tortuous fracture extracted less heat from the hot rock. This was related to the less efficient heat exchange caused by the channeling effect. The overall Nu number was higher in a rough fracture with a larger Re number due to the impact of the disturbance impact on the development of the boundary layer. Khalesi et al. [177] noted that HTC and pressure drop are influenced by the extensive variation of thermo-physical characteristics of s-CO_2_ near the critical point and along the pseudocritical region. The values of friction coefficient and Nu number vary along the channel as a result of the change in the velocity gradient and the wall heat flux. Operating pressure has a great impact on the pressure drop and heat transfer performance of the heat exchanger. Pressure drop and HT performance were remarkably higher for pressures away from the critical point than for pressures near the critical point. However, large variations of Nu number were not apparent for high operating pressures.

Taking into account the environmental friendliness of CO_2_ (global warming potential (GWP) index equal to 1) and its safety class A1 (according to the American Society of Heating, Refrigerating and Air-Conditioning Engineers (ASHRAE) standards [178]), CO_2_ has been recognized as the most promising refrigerant in applications where ammonia cannot be used. Various configurations of s-CO_2_ refrigeration were examined by Bellos and Tzivanidis [179]. For the evaporator temperature in the range from −35 to +5 °C, when changing the gas cooler outlet temperature from 35 to 50 °C, the system with the mechanical subcooling system was found to be the most efficient solution.

### 3.3. Molten Salts/Molten Salts NFs

Molten salts (MS) are another representative of novel HTFs. The term molten salt refers to the liquid prepared by melting inorganic salt or a mixture of salts. The mixing of salts led to a reduction in the melting point and simultaneously a steady rise in the high boiling temperature [180]. Typically, the operational temperature of molten salts is in the range of 250 to 1000 °C. Some of the main advantages of MS include good thermal conductivity, high stability, large specific heat capacity, low viscosity, environment friendliness, and low price. However, some of the MS heat transfer fluids possess a high melting point, even above 120  °C; thus, this may limit their application due to the risk of freezing. Another drawback is the high corrosivity of MS towards steel. Liu et al. [181] studied the risk of corrosion for stainless steel and alloys working with different MS HTFs. The authors observed the highest corrosion rate in NaCl-KCl-ZnCl_2_ MS, which was equal to 450 μm/year.

The heat transfer process of molten salts combines radiative, conductive, and convective heat transfer and due to the high viscosity, the dominant mode is radiative transfer with absorbing-scattering media [182]. A group of MS includes carbonates, fluorides, nitrides, and chlorides [183]. Usually used MS include Solar Salt (KNO_3_-NaNO_3_) and Hitec Salt (KNO_3_-NaNO_3_-NaNO_2_). Hitec Salt has a lower melting point but also lower stability than Solar Salt. Zou et al. [184] compared the thermophysical properties and thermal stability of Hitec Salt modified by the addition of CaNO_3_ with pure Hitec Salt and Solar Salt. A low melting point and a high decomposition point occurred for the Hitec salt with Ca(NO_3_)_2_. The appropriate operating temperature was 200–565 °C, which was higher than that of Hitec Salt and Solar Salt. Furthermore, the average specific heat and TC of the Hitec Salt with the Ca(NO_3_)_2_ additive were nearly 1520 J/(kg·K) and 0.655 W/(m·K), respectively, and MS presented improved heat transfer performance than Hitec salt and Solar Salt. Meanwhile, Vaka et al. [185] investigated the effect of different salt components, including NaNO_3_, KNO_3_, LiNO_3_, CsNO_3_, and Ca(NO_3_)_2_, on the melting point of the MS mixture. The authors observed that the sample with NaNO_3_: 4 wt%, KNO_3_: 22 wt%, LiNO_3_: 10.79 wt%, CsNO_3_: 44 wt%, and Ca(NO_3_)_2_: 19.20 wt% resulted in the lowest melting point of 61.40 °C. The results obtained show that with increasing KNO_3_, LiNO_3_, and CsNO_3_ in the salt mixture, the melting point decreased. Chen and Zhao [186] investigated the optimal ratio of Ca(NO_3_)_2_, NaNO_3_ and KNO_3_ in the MS mixture for heat transfer and energy storage applications. The researchers observed that the composition with a 32:24:44 wt% ratio for Ca(NO_3_)_2_, NaNO_3_ and KNO_3_, respectively, had the best thermal performance, with a specific heat capacity of 1700 and 1200 J/(kg·K) for the solid phase and liquid phase, respectively. This composition showed a relatively low melting point of about 80 °C, a low viscosity near zero at 200 °C and thermal conductivity in the range of 1 to 3 W/(m·K). Xu et al. [180] evaluated the potential of KCl–MgCl_2_ (molar ratio 32:68) application as a HTFs and TES material. The authors applied the MS at a temperature of up to 800 °C. The specific heat capacity in the liquid state was between 990–1013 J/(kg·K), while the TC was between 0.465 and 0.424 W/(m·K) at temperatures of 450–800 °C. However, the average melting point was relatively high and equal to about 424 °C. The initial treatment of MS led to the elimination of water and oxygen and contributed to the lower corrosivity. Trablesi et al. [187] compared the use of synthetic oil, therminol and molten salt (NaNO_3_-KNO_3_). In the power plant with MS applied as HTF, the overall energy efficiency increased by about 6%, compared to therminol. Meanwhile, the approximate decrease in cost was about 20%.

In addition, an interesting combination is molten salt nanofluids (MSNFs). As for other nanofluids, the major drawbacks include the increase in viscosity and problems with stability. These NFs offer greater compatibility for corrosion resistance and prevention in steel at higher temperatures. The NPs dispersed in the molten salt encourage the creation of an electrical double layer on the surface of the NPs. Ordinarily, silica or alumina NPs possess a hydroxyl group attached to their surface and thus have a negatively charged surface. The molten salt mixture possesses a positive charge, with different values of the zeta potential. One salt will have a higher zeta potential than the other, and therefore will be more attracted to negatively charged NPs than the other salt and start to assemble around it [188]. Repulsion between the electric double layers considerably reduced the agglomeration between NPs, and consequently, NPs disperse [189]. Xiong et al. [190] used Solar Salt with the addition of 1% of SiO_2_ NPs. The TC enhancement was about 52% for the sample prepared by the high-temperature melting method with 90 min of mixing, compared to the base MS. However, the melting point decreased by about 7 °C and the specific heat increased by about 46%. Li et al. studied the effect of introduction of 10 wt% of SiO_2_ NPs to Solar Salt on the thermal properties. The researchers observed an increase in TC of up to 54.5%. Furthermore, the authors proved that the improvement of TC may be assigned to the enhanced probability and frequency of ion collision, indicated by the change in potential energy. Ying et al. [191] investigated the heat transfer potential of Al_2_O_3_/Hitec. For 0.063 wt% of Al_2_O_3_ NPs, the greatest heat transfer performance was recorded, with an increase in HTC by 7.29% and Nu number by 6.9%. The authors observed that Al_2_O_3_/Hitec MSNFs markedly decreased the peak temperature of the outer and inner heated surface of the tube wall. They also concluded that at a low NP content, the specific heat capacity mostly affected the heat transfer. Additionally, Wei et al. [192] proposed the use of Solar Salt with MgO NPs. The NPs content was in the range of 2.5–10 wt%. The researchers concluded that the optimal amount of NPs was 5% with an increase in specific heat capacity of 11.9% and thermal conductivity of up to 62.5%. Meanwhile, they did not observe the remarkable growth in viscosity (up to 0.6%).

### 3.4. Ionic Liquids/Ionanofluids

One of the important groups of novel HTFs is also ionic liquids (ILs). Ionic liquids are salts composed only of ions, which exist in the liquid form at a temperature below 100 °C [193]. Commonly, they consist of organic cations and organic/inorganic anions. The cations are, e.g., azolium, phosphonium, pyridinium, pyrrolidinium, and alkylammonium. Inorganic anions include, for instance, halides, nitrates, perchlorates, sulfates, and azides. Meanwhile, organic anions may include, for example, benzoates, sulfacetamides, alkylcarbonates, and organic carboxylates [194]. As opposed to molten salts, which usually operate at high temperatures and are highly corrosive and viscous materials, the ILs can be liquids at very low temperatures, as low as –96 °C, and possess very low viscosity and corrosivity [193]. ILs show a low freezing point, a wide liquid range, non-flammability, high thermal stability, and very low vapor pressure [195]. Wadekar [34] characterized the potential application of [bmim][Tf2N] in a plate heat exchanger, and in a shell-and-tube heat exchanger. The author compared the thermal properties of [bmim][Tf2N] with the Dowtherm. Dowtherm showed the higher HTC and the higher pressure drop than IL. Oster et al. [196] described the thermal properties of trihexyl(tetradecyl)phosphonium acetate ([AcO]^−^), butanoate ([ButO]^−^), hexanoate ([HexO]^−^), octanoate ([OctO]^−^), and decanoate ([DecO]^−^) ILs and their mixtures with water. Density and heat capacity decreased with the growth of the anion chain length, and the authors observed the same trend for IL-water mixtures. The density of pure ILs was lower than the density of water (10–13%). The TC of ILs decreased to [P14,6,6,6][HexO], while further growth was observed in the alkyl chain length. For water–IL mixtures, the enhancement in TC was between 15.24% and 18.59%, compared to pure ILs. The author concluded that the major limitation of ILs’ application as HTFs is the relatively high price, thus the preparation of water–ILs mixtures may remarkably reduce the costs.

Commonly proposed solutions to improve the thermal characteristics of ionic liquids include the addition of various nanoparticles and the formation of ionanofluids, also called as nanoparticles, which enhance ionic liquids. Paul et al. [197] compared the thermal properties of the ionic liquid [C4mim][NTf2] and an ionanofluid with Al_2_O_3_ NPs. INFs showed an enormous enhancement of shear viscosity with the addition of a small amount of NP. TC increased with NPs concentration, and the maximum enhancement of up to 11% occurred for 2.5 wt% Al_2_O_3_. Furthermore, the heat capacity of INFs increased significantly by up to 62% with 2.5 wt% Al_2_O_3_. Hosseinghorbani et al. [198] added graphene oxide to [Bmim][NTf2] as a HTF in a concentrated solar power plant. The authors observed that the graphene oxide INFs presented excellent stability for up to 2 weeks. Thermal conductivity and specific heat capacity grew by 6.5% and 42%, respectively, at 2 wt.% of graphene oxide NPs. However, the greatest HTC enhancement of 7.2% was achieved for 0.5 wt.% of graphene oxide NPs. In the study presented by Jóźwiak et al. [146], multiwalled carbon nanotubes were introduced to the [C2C1im][SCN] and applied as a working fluid in the heat exchanger. The highest improvement in average convective HTC by 48.1% was recorded for 0.25 wt% of MWCNT. However, the pressure drop increased by about 55.2% in comparison to pure ILs. Thus, the application of INFs may require the installation of much more powerful delivery pumps. Despite this, the performance evaluation criteria reached a value above 1. Das et al. [199] incorporated the MXene NPs into an [MMIM][DMP]/water(20:80 v/v) mixture. INFs showed good stability and the highest improvement of TC was observed by 47% for 0.2 wt% of NPs. The authors compared the results for MXene/water-INFs with the results for MXene/palm oil and Al_2_O_3_/water NFs. HTC increased by 12.6% and 2% for aqueous INFs compared to Al_2_O_3_/water and MXene/palm oil, respectively.

### 3.5. Nano- and Micro-Encapsulated Phase Change Materials

The next group of novel heat transfer materials comprises encapsulated phase change materials (EPCM). These EPCM materials can absorb and release energy during the phase change transition period, charging and discharging processes at a specific fusion temperature [200]. EPCM materials can be applied in the lower, medium, and higher temperature range (even from −20 up to 200 °C), depending on the properties of the components [201]. Based on the size of the EPCM, researchers classified them as micro-encapsulated PCM (MEPCM) and nano-encapsulated PCM (NEPCM) with micrometric and nanometric size, respectively. Further reduction in the EPCM size led to an increase in the thermal transportation capacity. Encapsulated PCM includes inorganic–inorganic, organic–organic, and inorganic–organic combinations. In the EPCM construction, the shell seals the PCM core. Examples of cores include paraffin, polyethylene glycol, n-octadecane, gallium, bismuth, CaCl_2_ hexahydrate, and many more. However, for example, graphene and its derivatives, silica, and polymers, e.g., melamine-formaldehyde resin, polymethyl methacrylate (PMMA), polyurethane (PU), and polystyrene (PS) may comprise the shell. There are many significant advantages related to the application of encapsulated PCM, such as the prevention of PCM leakage, and excellent thermal transport capability because of the increase in the heat transfer surface area. The presence of encapsulated PCM in the base fluid can improve its thermal conductivity [202]. Chananipoor et al. [203] characterized EPCM suspension composed of n-dodecanol as the core and modified PMMA with graphene oxide as the shell in a double pipe heat exchanger. The authors found that in the turbulent tubular flow of the NEPCM suspension in water, the inlet temperature and the mass fraction of the NEPCM have the greatest impact on the heat transfer performance. The experimental results show that the proposed NPCMs with content equal to 14% could improve heat transfer for thermal systems compared to pure water. Ghalambaz et al. [204] studied the effect of the addition of ECPM to water, with nonadecan as the inner part and polyurethane as the outer layer. Introducing NEPCM led to an increase in heat transfer of 13%. The phase change of the NEPCM caused an increase of up to about 28% in heat transfer. Nomura et al. [205] described the properties of MEPCM composed of Al-Si microspheres as a core and Al_2_O_3_ as a shell for heat storage and transfer. The heat capacity was about 233 J/g, and the composite showed outstanding stability up to 300 heating and cooling cycles. Yang et al. [200] characterized the paraffin@graphene MEPCM. The thermal conductivity of MEPCM was up to 0.418 W/(m·K), which was 2.34 times higher than paraffin. For the MEPCM with a paraffin core content greater than 99%, the phase change latent heat was 232.4 J/g, and therefore was higher than for pure paraffin.

### 3.6. Summary of Environmental, Technical, and Economic Aspects of Novel HTFs

Table 2 summarizes the types of novel heat transfer fluids with a detailed comparison of the environmental, technical, and economic aspects of their application. Due to the focus on competing HTFs with respect to conventional HTFs, all the examples presented in Table 2 are widely available.

## 4. Application, Advantages, and Limitations of Conventional and Novel HTFs

The field of HTFs is developing very quickly. This is dictated by the tremendous demand coming from the industry. Nowadays, HTFs find applications in almost every sector of life. Heat transfer fluids are used as intermediate fluids in processes where cooling or heating is required to reach the desired temperature. In some devices with compact size, shape, or weight, conventional heat transfer fluids such as air or water are not efficient enough, and thus the application of novel transfer fluids with improved thermal characteristics is highly recommended. Table 3 summarizes examples of their employment described in the literature. Furthermore, the major advantages and limitations of each HTF have been considered. Almost all listed fluids can be applied in heat exchangers, being a part of various systems and technologies. One of the key technologies for HTFs application is the concentrated solar power system. In this process, HTFs transfer heat from the receiver to the steam generator. Additionally, HTF may be stored in an insulated tank for power generation in case there is no access to sunlight. Typically, the most favorable HTFs for CSP plants due to their high working temperature and good heat capacity are molten salts [16]. Another example describes the use of HTFs in a secondary loop refrigeration system in coolers, freezers, residential air conditioners or mobile air conditioners [209]. In this case, the most promising candidates are nanofluid refrigerants or encapsulated PCM. For instance, the HTFs are applicable in the cooling of advanced electronic components. Due to the minimal size of electronic devices, a high heat dissipating rate is required [84]. Currently, the most developed group of HTFs in this sector is nanofluids. Furthermore, HTFs may be successfully used to recover heat from a low-temperature exhaust in an organic Rankine cycle. The HTF receives heat from an exhaust source. Subsequently, a second working fluid is vaporized, which flows through a turbine to generate electricity. In this case, nanofluids were also proposed in many studies [210]. Furthermore, an important sector in the use of HTFs is geothermal systems. Nowadays, supercritical CO_2_ has replaced water as a HTF in these systems [211]. In another paper, the application of methanol, ethanol, and propylene glycol was proposed [212].

Table 4 contains the percentage change in the thermophysical and physicochemical properties of selected nanofluids compared to the base fluids. First of all, the addition of nanoparticles to the base fluid does not result in either narrowing or extending the temperature range of the HTF operation, both in the case of water-based and EG/water-based nanofluids. Moreover, the presence of NPs has a weak effect on physicochemical properties, such as density and specific heat. However, a significant influence on the viscosity is observed, as in the case of water-based nanofluids, it is usually much higher as compared to the base fluid (up to 150% higher for 20% n-dodecanol/PMMA/GO/water NF). However, the addition of NPs to the EG/water mixture reduces the dynamic viscosity. Otherwise, the presence of NPs affects the thermophysical properties, as a significant increase in the thermal conductivity is observed. This means that nanofluids are better heat conductors than pure base fluids. The change in the demonstrated physicochemical and thermophysical properties results in a change in the value of the Prandtl number. Water-based NFs usually have an increased number of Pr in relation to pure water, while in the case of EG/water-based NFs, a decrease in the value of the Pr number is observed.

## 5. Conclusions and Future Recommendations

In this review, major groups of HTFs were described considering their classification as conventional or novel materials. State-of-the-art analysis led to the conclusion that conventional fluids are being displaced by novel materials, mainly nanofluids.

Currently, NFs are the most rapidly developing and expanding group of HTFs. Their beneficial features include the wide range of base fluids and incorporated nanoparticles, which leads to a tunable composition depending on the purpose. From the economic and environmental points of view, the most favorable NFs are water-based NFs. Even with the introduction of higher-priced NPs such as graphene, CNT, or MXene, adding them in such a small amount will not remarkably increase the costs. Generally, the cost of NFs depends on the price of the base fluid. The application of hybrid NFs with synergistic effect is a suitable solution, taking into account the economic point of view and the efficiency of the processes. However, the main limitation with respect to the application of water-based NFs is the fact that they will operate satisfactorily up to 100 °C. Moreover, it is important to properly select the content of NPs, because too high an amount will lead to the agglomeration and sedimentation and, therefore, will limit the long-term use of NFs. Important aspects that should be further investigated and developed may be the use of surfactants or functionalization of NPs as one way to prevent the destabilization of the suspension. Proper methods should be proposed considering the types of nanoparticles as well as characteristics of base fluids. Table 5 summarizes the most important advantages and disadvantages of nanofluids in terms of environmental, technical, and economic aspects.

Ultra-low and ultra-high temperature applications require the utilization of materials that are thermally stable and able to operate in such harsh conditions. Here, the solution may be the use of molten salts or supercritical CO_2_. The main advantages of supercritical CO_2_ utilization are their non-toxic and non-corrosive nature, and wide operational range. In contrast, molten salts are highly corrosive materials.

## Figures and Tables

**Table 1:** Environmental, technical, and economic aspects comparison of examples of conventional heat transfer fluids.

Fluid	Environmental Aspects		Technical/Efficiency Aspects						Economic Aspects
	Corrosivity	Toxicity	Temperature Range (°C)	ρ (kg/m^3^)	c (J/(kg·K))	TC (W/(m·K))	η (mPa·s)	Pr (-)	Price ($/kg)
**air**	No	No	+540 to 1090	1.293 (0 °C) 1.128 (40 °C) 1.000 (80 °C)	1004 (0 °C) 1004 (40 °C) 1006 (80 °C)	0.024 (0 °C) 0.028 (40 °C) 0.031 (80 °C)	0.017 (0 °C) 0.019 (40 °C) 0.021 (80 °C)	0.71 (0 °C) 0.68 (40 °C) 0.68 (80 °C)	0
**fluoroalkanes**	No	No	−100 to +150	1840 (0 °C) 1800 (20 °C)	1000 (0 °C) 1050 (20 °C)	0.06 (0 °C) 0.06 (20 °C)	2 (0 °C) 1.3 (20 °C)	33.3 (0 °C) 22.8 (20 °C)	5 (TCE)
**water**	Yes	No	0 to +100	1000 (0 °C) 992 (40 °C) 972 (80 °C)	4222 (0 °C) 4175 (40 °C) 4195 (80 °C)	0.558 (0 °C) 0.633 (40 °C) 0.673 (80 °C)	1.792 (0 °C) 0.656 (40 °C) 0.357 (80 °C)	13.56 (0 °C) 4.33 (40 °C) 2.23 (80 °C)	0.01
**aliphatic hydrocarbons**	Yes	No	−60 to +150	815 (−50 °C)	1880 (−50 °C)	0.115 (−50 °C)	15 (−50 °C)	245.2 (−50 °C)	2–4 (isoparaffinic hydrocarbons)
**aromatic hydrocarbons**	Yes	Yes	−70 to +260	920 (−50 °C)	1636 (−50 °C)	0.143 (−50 °C)	3.9 (−50 °C)	44.6 (−50 °C)	2.5–3 (diethylbenzene)
**ethylene glycol** **(41 wt% aqueous solution)**	Yes	Yes	−20 to +100	1069 (−20 °C) 1062 (0 °C)	3320 (−20 °C) 3405 (0 °C)	0.391 (−20 °C) 0.406 (0 °C)	15.6 (−20 °C) 5.95 (0 °C)	132.5 (−20 °C) 49.9 (0 °C)	1.5
**propylene glycol** **(44 wt% aqueous solution)**	Yes	No	−20 to +100	1053 (−20 °C) 1045 (0 °C)	3620 (−20 °C) 3640 (0 °C)	0.362 (−20 °C) 0.373 (0 °C)	62 (−20 °C) 13.9 (0 °C)	620 (−20 °C) 135.6 (0 °C)	3
**silicone oil**	No	No	−100 to +260	927 (−50 °C) 900 (0 °C) 890 (20 °C)	1625 (−50 °C) 1700 (0 °C) 1750 (20 °C)	0.125 (−50 °C) 0.110 (0 °C) 0.100 (20 °C)	6.9 (−50 °C) 2 (0 °C) 1.2 (20 °C)	89.7 (−50 °C) 30.9 (0 °C) 21 (20 °C)	3–5
**paraffinic oil**	No	No	+30 to +300	721 (300 °C)	2436 (300 °C)	~0.1 (300 °C)	1.09 (300 °C)	26.6 (300 °C)	2–5
**eutectic mixture of biphenyl/diphenyl oxide**	No	Yes	+12 to +400	849 (300 °C)	1930 (300 °C)	~0.01 (300 °C)	0.59 (300 °C)	113.9 (300 °C)	100

**Table 2:** Environmental, technical, and economic aspects comparison of examples of novel heat transfer fluids.

Fluid	Environmental Aspects		Technical Aspects						Economic Aspects	Ref.
	Corrosivity	Toxicity	Temperature Range (°C)	ρ (kg/m^3^)	c (J/kg·K)	TC (W/m·K)	η (mPa·s)	Pr (-)	Estimated Price($/kg)	
**0.6 vol% Ni/water**	Yes	No	0 to +100	1045 (20 °C) 1035 (50 °C)	4160 (20 °C) 4158 (50 °C)	0.72 (20 °C) 0.84 (50 °C)	1.5 (20 °C) 0.85 (50 °C)	8.67 (20 °C) 4.21 (50 °C)	0.13	[132]
**0.04 vol% ZnO/H_2_O–EG(50:50 *v*/*v*)**	Yes	Yes	−20 to +100	1002 (60 °C)	4016 (60 °C)	0.708 (60 °C)	0.89 (60 °C)	5.048 (60 °C)	0.76	[206]
**0.16% Cu-Al(4:1) LDH/water**	Yes	No	0 to +100	n.d	n.d	0.68 (30 °C)	1.35 (30 °C)	n.d	n.d.	[140]
**0.05 vol% graphene/water**	Yes	No	0 to +100	998.5 (20 °C) 992.5 (45 °C)	4060 (20 °C) 4075 (45 °C)	0.675 (20 °C) 0.78 (45 °C)	1.12 (20 °C) 0.72 (45 °C)	6.736 (20 °C) 3.76 (45 °C)	0.04	[152]
**0.1 wt% MWCNT/CuO/water**	Yes	No	0 to +100	998.8 (20 °C) 992.8(40 °C)	4178(20 °C) 4175 (40 °C)	0.54 (20 °C) 0.76 (40 °C)	1.02 (20 °C) 0.66 (40 °C)	7.89 (20 °C) 3.63 (40 °C)	0.07	[207]
**0.04 vol%** **f-CNT/rGO/water**	Yes	No	0 to +100	997.5 (25 °C) 986.0 (55 °C)	n.d	0.65 (25 °C) 0.80 (55 °C)	1.1 (25 °C) 0.6 (55 °C)	n.d	n.d.	[169]
**supercritical CO_2_**	Yes	No	−73 to +1000	800 (25 °C) 300 (50 °C) 190 (100 °C) *p* = 10 MPa	3000 (25 °C) 4000 (50 °C) 1500 (100 °C) *p* = 10 MPa	0.085 (25 °C) 0.04 (50 °C) 0.03 (100 °C) *p* = 10 MPa	0.07 (25 °C) 0.025 (50 °C) 0.025 (100 °C) *p* = 10 MPa	2.47 (25 °C) 2.5 (50 °C) 1.3 (100 °C) *p* = 10 MPa	n.d.	[174]
**Solar Salt**	Yes	No	+220 to +550	1900 (300 °C) 1775 (500 °C)	1495 (300 °C) 1800 (500 °C)	0.5 (300 °C) 0.54 (500 °C)	3.0 (300 °C) 1.25 (500 °C)	8.97 (300 °C) 4.17 (500 °C)	0.49	[208]
**0.5 wt% SiO_2_/Hitec**	Yes	No	+142 to +450	n.d	1890 (200 °C) 2000 (300 °C)	0.475 (200 °C) 0.525 (300 °C)	2.25 (200 °C) 1.75 (300 °C)	8.95 (200 °C) 6.67 (300 °C)	0.93	[189]
**20% n-dodecanol/PMMA/GO/water**	No	No	0 to +100	949.4 (20 °C)	3650 (20 °C)	0.5 (20 °C)	2.49 (20 °C)	18.18 (20 °C)	n.d.	[203]
**Ionic liquid** **([bmim][Tf2N])**	Yes	No	+25 to +200	1429 (25 °C) 1354 (100 °C) 1254 (200 °C)	1252 (25 °C) 1430 (100 °C) 1667 (200 °C)	0.1271 (25 °C) 0.1219 (100 °C) 0.1149 (200 °C)	41.0 (25 °C) 8.1 (100 °C) 1.5 (200 °C)	403.87 (25 °C) 95.02(100 °C) 21.76 (200 °C)	n.d.	[34]

n.d.—no data.

**Table 3:** Examples of various heat and coolant transfer fluids applications.

Fluid	Applications	Advantages	Limitations	Ref.
air	- Thermal energy storage - Photovoltaic systems	- Freely available - Environmental friendly - Non-vulnerable to freezing and boiling	- Lower thermal conductivity and volumetric heat capacity	[41,46,47]
water	- Parabolic trough collectors - Thermal energy storage	- Low cost - High specific heat - Non-toxicity - Low viscosity	- Corrosive nature - Freezing below 0 °C	[56,213]
nanofluids	- Electronic devices - Solar collectors - Car radiators - Spray cooling - Engines cooling - Chilled water air conditioning - Refrigeration systems - Jet impingement cooling	- Higher thermal conductivity and heat capacity - Various choice of base fluids and nanoparticles	- Problems with stability - Possible fouling effect - Higher viscosity and pressure drop	[104,130,154,206,214,215,216,217,218]
supercritical CO_2_	- Solar collectors - Thermal energy storage - District heating - Rankine and Brayton cycles - Concentrated Solar Power Plants	- Inexpensive - Non-flammable - Non toxic - Easier to compress than steam - Less corrosive than steam - Operational temperature range from −73 to 1000 °C	- Higher initial capital investments - Dramatic decrease in density limits the capability to be applied as a storage medium	[113,176,219,220,221]
molten salts	- Concentrated solar power plants - Thermal energy storage - Jet impingement cooling	- High operation temperatures - Good thermal conductivity - High stability - Large specific heat capacity	- Freezing at high temperatures - High corrosivity	[185,222,223,224,225]
EPCM	- Photovoltaic systems - Thermal energy storage - Air conditioning - Domestic heat pump - Electronic devices	- High thermal conductivity - Long-term chemical stability - Non-toxicity - Non-flammable - Non-corrosive	- Phase segregation and supercooling - High cost of encapsulation	[201,203,226,227,228]
ionic liquids	- Refrigeration systems - Solar collectors - Concentrated Solar Power Plants	- Thermal stability - Non-volatility and explosion safety - Ionic conductivity - High heat capacity	- High cost - Corrosivity	[198,199,229]

**Table 4:** Percentage change in thermophysical and physicochemical properties of selected nanofluids compared to the base fluid.

Fluid	Temperature Range (°C)	Percentage Change in Parameter Compared to the Base Fluid				
		ρ (kg/m^3^)	c (J/kg·K)	TC (W/m·K)	η (mPa·s)	Pr (-)
**Water-based nanofluids**						
**0.6 vol% Ni/water**	0 to +100	+4.7% (20 °C) +4.8% (50 °C)	−0.47% (20 °C) −0.47% (50 °C)	+20.6% (20 °C) +29.8% (50 °C)	+49.3% (20 °C) +54.8% (50 °C)	+23.2% (20 °C) +18.6% (50 °C)
**0.16% Cu-Al(4:1) LDH/water**	0 to +100	n.d.	n.d.	+10.6% (30 °C)	+68.6% (30 °C)	n.d.
**0.05 vol% graphene/water**	0 to +100	+0.05% (20 °C) +0.3% (45 °C)	−2.9% (20 °C) −2.4% (45 °C)	+13.1% (20 °C) +21.9% (45 °C)	+11.4% (20 °C) +19.4% (45 °C)	−4.3% (20 °C) −4.6% (45 °C)
**0.1 wt% MWCNT/CuO/water**	0 to +100	+0.08% (20 °C) +0.08% (40 °C)	−0.05% (20 °C) 0% (40 °C)	−9.5% (20 °C) +20.1% (40 °C)	+1.5% (20 °C) +0.6% (40 °C)	+12.1% (20 °C) −16.2% (40 °C)
**0.04 vol%** **f-CNT/rGO/water**	0 to +100	+0.05% (25 °C) +0.05% (55 °C)	n.d.	+7.3% (25 °C) +22.5% (55 °C)	+22.2% (25 °C) +17.9% (55 °C)	n.d.
**20% n-dodecanol/PMMA/GO/water**	0 to +100	−4.9% (20 °C)	−12.7% (20 °C)	−16.2% (20 °C)	+147.8% (20 °C)	+158.2% (20 °C)
**Water-EG-based nanofluids**						
**0.04 vol% ZnO/H_2_O–EG(50:50 v/v)**	−20 to +100	−8.4% (20 °C)	+14.9% (20 °C)	+68.2% (20 °C)	−88.8% (20 °C)	−92.4% (20 °C)

n.d.—no data.

**Table 5:** Summary of advantages and disadvantages of nanofluids in terms of environmental, technical and economic aspects.

Approach	Advantages	Disadvantages
Environmental	Most of the currently proposed NFs are non-toxic.	The use of NFs based on nanoparticles does not eliminate the problem of corrosivity of HTFs.
Technical	NFs do not have a narrower operating temperature range. NFs are better heat conductors than base fluids, as the addition of NPs results in an increase in TC.	NFs do not have a wider operating temperature range. Very often NFs have higher dynamic viscosity compared to base fluids, which is a problem from the installation point of view. Too higher amount of NPs will lead to the agglomeration and sedimentation, and therefore limits long-term use of NFs.
Economical	There are a wide range of base fluids and incorporated nanoparticles, and thus led to get a tunable composition depending on the purpose. Even with the introducing of higher-priced NPs, adding them in such a small amount will not remarkably rise the costs. There are many low-cost solutions.	Some NFs, despite more favorable physicochemical and thermophysical properties, are too expensive to be implemented.

## Data Availability

Data available in a publicly accessible repository as well as in a publicly accessible repository that does not issue DOIs. All data referred to in this paper are accessible according to the list of references.

## Abbreviations

[Bmim][NTf2]	1-butyl-3-methylimidazolium-bis(trifluoromethylsulfonyl)imide
[bmim][Tf2N]	1-butyl-3-methylimidazolium bis[(trifluoromethyl)sulfonyl]imide
[C2C1im][SCN]	1-ethyl-3-methylimidazolium thiocyanate ionic liquid
[C4mim][NTf2]	1-butyl-3-methylimidazolium bis{(trifluoromethyl)sulfonyl}imide
[MMIM][DMP]	1,3-dimethylimidazolium dimethyl-phosphate
ASHRAE	American Society of Heating, Refrigerating and Air-Conditioning Engineers
c	specific heat [J/(kgꞏK)]
CHF	critical heat flux [MW/m^2^]
CNT	carbon nanotubes
CSP	Concentrating Solar Power
Da	Damköhler numbers [–]
DPHE	double pipe heat exchanger
EG	ethylene glycol
EPCM	encapsulated phase change materials
f-ZnO NFs	functionalized ZnO nanofluids
GHE	geothermal heat exchanger
GNPs	graphene nanoparticles
GO	graphene oxide
GWP	Global Warming Potential
HE	heat exchanger
HTC	heat transfer coefficient [W/(m^2^ꞏK)]
HTF	heat transfer fluid
ILs	ionic liquids
INFs	ionanofluids
LDH	layered double hydroxides
MEPCM	micro-encapsulated phase change materials
MS	molten salts
MSNFs	molten salt nanofluids
MWCNT	multi-walled carbon nanotubes
NEPCM	nano-encapsulated phase change materials
NFs	nanofluids
NPs	nanoparticles
Nu	Nusselt number [–]
ORC	Organic Rankine Cycle
PCM	phase change materials
PG	propylene glycol
PMMA	polymethyl methacrylate
Pr	Prandtl number [–]
PS	polystyrene
PU	polyurethane
Ra	Rayleigh number [–]
Re	Reynolds number [–]
rGO	reduced graphene oxide
s-CO_2_	supercritical CO_2_
SWCNT	single-walled carbon nanotubes
TC	thermal conductivity [W/(mꞏK)]
TCE	trichloroethylene
TES	thermal energy storage
η	dynamic viscosity [mPaꞏs]
ρ	density [kg/m^3^]
