# Correction: Czaplicka et al. Promising Nanoparticle-Based Heat Transfer Fluids—Environmental and Techno-Economic Analysis Compared to Conventional Fluids. *Int. J. Mol. Sci.* 2021, *22*, 9201

**DOI:** 10.3390/ijms26188851

**Published:** 2025-09-11

**Authors:** Natalia Czaplicka, Anna Grzegórska, Jan Wajs, Joanna Sobczak, Andrzej Rogala

**Affiliations:** 1 Department of Process Engineering and Chemical Technology, Faculty of Chemistry, Gdansk University of Technology, Narutowicza 11/12, 80-233 Gdansk, Poland; anna.grzegorska@pg.edu.pl (A.G.); andrzej.rogala@pg.edu.pl (A.R.); 2 Institute of Energy, Faculty of Mechanical Engineering and Ship Technology, Gdansk University of Technology, Narutowicza 11/12, 80-233 Gdansk, Poland; jan.wajs@pg.edu.pl; 3 Research and Development Joanna Sobczak, Różnowo 8, 14-240 Susz, Poland; joasobczak@outlook.com

In the original publication [1], the funding from the Minister of Science and Higher Education, Poland, contract no. 5232/SBP 2014-2020/2022/2, was not included, and no information on this was given in the Funding Section, stating that “This research received no external funding”. However, it was mentioned in the Acknowledgments Section that “This manuscript was prepared within a scope of a Baltic Smart Asset Management (BSAM) project, co-financed by the Interreg South Baltic Program 2014–2020”.

Corrections have been made in the Acknowledgments and Funding sections.

The information in the Acknowledgment Section was deleted.

The corrected statement in the Funding Section is as follows:

“This manuscript was prepared within the scope of a Baltic Smart Asset Management (BSAM) project, co-financed by the Interreg South Baltic Program 2014–2020, project number STHB.02.02.00-SE-0149/18, and published as part of an international project co-financed by the program of the Minister of Science and Higher Education entitled “PMW” in 2021-2022-Baltic Smart Asset Management (BSAM); contract no. 5232/SBP 2014-2020/2022/2”.

The authors state that the scientific conclusions are unaffected. This correction was approved by the Academic Editor. The original publication has also been updated.
